# Source apportionment of PM_10_ and health risk assessment related in a narrow tropical valley. Study case: Metropolitan area of Aburrá Valley (Colombia)

**DOI:** 10.1007/s11356-023-26710-1

**Published:** 2023-04-05

**Authors:** Carlos Ramos-Contreras, María Piñeiro-Iglesias, Estefanía Concha-Graña, Joel Sánchez-Piñero, Jorge Moreda-Piñeiro, Amaya Franco-Uría, Purificación López-Mahía, Francisco Molina-Pérez, Soledad Muniategui-Lorenzo

**Affiliations:** 1 Department of Chemistry, Faculty of Sciences, Grupo Química Analítica Aplicada (QANAP), University Institute of Research in Environmental Studies (IUMA), University of A Coruña, Campus de A Coruña, S/N. 15071, A Coruña, Spain; 2 Grupo de Investigación en Gestión y Modelación Ambiental (GAIA), Escuela Ambiental, Facultad de Ingeniería, Universidad de Antioquia UdeA, Calle 70 No. 52-21, Medellín, Colombia; 3 Dept. of Chemical Engineering, School of Engineering, University of Santiago de Compostela, 15782 Santiago de Compostela, Spain

**Keywords:** Particulate matter, Metal(oid)s, δ^13^C carbon isotope ratios, Polycyclic aromatic hydrocarbons, Source apportionment, Health risk assessment

## Abstract

This study investigates spatio-temporal variations of PM_10_ mass concentrations and associated metal(oid)s, δ^13^C carbon isotope ratios, polycyclic aromatic hydrocarbons (PAHs), total organic carbon (TOC) and equivalent black carbon (eBC) concentrations over a half year period (from March 2017 to October 2017) in two residential areas of Medellín (MED-1 and MED-2) and Itagüí municipality (ITA-1 and ITA-2) at a tropical narrow valley (Aburrá Valley, Colombia), where few data are available. A total of 104 samples were analysed by using validated analytical methodologies, providing valuable data for PM_10_ chemical characterisation. Metal(oid)s concentrations were measured by inductively coupled plasma mass spectrometry (ICP-MS) after acid digestion, and PAHs concentrations were measured by Gas Chromatography-Mass Spectrometry (GC–MS) after Pressurised Hot Water Extraction (PHWE) and Membrane Assisted Solvent Extraction (MASE). Mean PM_10_ mass concentration ranged from 37.0 µg m^−3^ to 45.7 µg m^−3^ in ITA-2 and MED-2 sites, respectively. Al, Ca, Mg and Na (from 6249 ng m^−3^ for Mg at MED-1 site to 10,506 ng m^−3^ for Ca at MED-2 site) were the major elements in PM_10_ samples, whilst As, Be, Bi, Co, Cs, Li, Ni, Sb, Se, Tl and V were found at trace levels (< 5.4 ng m^−3^). Benzo[g,h,i] perylene (BghiP), benzo[b + j]fluoranthene (BbjF) and indene(1,2,3-c,d)pyrene (IcdP) were the most profuse PAHs in PM_10_ samples, with average concentrations of 0.82–0.86, 0.60–0.78 and 0.47–0.58 ng m^−3^, respectively. Results observed in the four sampling sites showed a similar dispersion pattern of pollutants, with temporal fluctuations which seems to be associated to the meteorology of the valley. A PM source apportionment study were carried out by using the positive matrix factorization (PMF) model, pointing to re-suspended dust, combustion processes, quarry activity and secondary aerosols as PM_10_ sources in the study area. Among them, combustion was the major PM_10_ contribution (accounting from 32.1 to 32.9% in ITA-1 and ITA-2, respectively), followed by secondary aerosols (accounting for 13.2% and 23.3% ITA-1 and MED-1, respectively). Finally, a moderate carcinogenic risk was observed for PM_10_-bound PAHs exposure via inhalation, whereas significant carcinogenic risk was estimated for carcinogenic metal(oid)s exposure in the area during the sampling period.

**Supplementary Information:**

The online version contains supplementary material available at 10.1007/s11356-023-26710-1.

## Introduction

Several adverse health effects have been associated with atmospheric particulate matter (PM) exposure by epidemiological studies decades ago (Manisalidis et al. 2020; Pope and Dockery 2006), resulting in increased mortality and morbidity rates mainly due to respiratory and cardiovascular diseases, including lung cancer (Chen and Hoek 2020; Mueller et al. 2021; Wang et al. 2020). Due to the spatial–temporal variability of atmospheric particles, PM may encompass many associated pollutants (both being constituents of particles or being adsorbed on their surfaces) which are potential contributors to PM adverse health effects as they can be potentially absorbed to bloodstream after inhalation (Arias-Pérez et al. 2020; Lu et al. 2015; Mousavi et al. 2017).

As posing a great threat to human health, air quality policies should entail PM source apportionment studies to identify possible PM sources and their chemical profile since they would lead to a better assessment of PM-associated health risks. Considering that, it would allow the development and implementation of effective mitigation policies and strategies to protect human health from PM pollution (Hopke 2008). Measurement of PM elemental and organic composition together with PM properties such as elemental and organic carbon would be of great interest as they can be used as source tracers for PM apportionment studies (Hsu et al. 2016; Liu et al. 2020). In addition, study of parameters such as stable carbon isotope ratio (^13^C/^12^C expressed as a δ^13^C) in PM samples can support apportionment studies to identify potential anthropogenic aerosol sources (road traffic or industrial emissions) in typical urban environments (Buczyńska et al. 2013; Kunwar et al. 2018, 2016; Morera-Gómez et al. 2021; Widory 2006).

Exposure to atmospheric pollutants and their health impacts will be influenced by meteorological conditions and topography of the areas studied. On this basis, PM exposure could potentially be increased in settlements located in narrow valleys where local conditions do not allow an adequate diffusion such as Aburrá Valley (Medellín Metropolitan Area, Antioquia, Colombia), located in a mountainous area in which some municipalities of Medellín Metropolitan Area are settled. Hence, the study of atmospheric pollutants transport and transformation require comprehensive knowledge of related meteorological phenomena (such as wind speed and direction, temperature, precipitation and solar radiation). Epidemiological studies conducted in the Metropolitan Area of Aburrá Valley (ANVA) show that the burden of disease attributable to PM exposure represented about 9.2% of total deaths during 2011, whereas around 72% of the mortality due to air pollution in Medellín was associated with Aburrá Valley area (AMVA 2017a). According to the ANVA’s air quality network, vehicular exhaust emissions were the major source of PM in the area, whilst local studies concerning mountain meteorology shown that daytime winds rise during dry days along the valley, entering by the northeast branch (Adarve and Molina 1984). Additionally, significant thermo-dependent diffusion processes at ground level have been reported to cause important variations of atmospheric boundary layer’s (ABL) height in the area, ranging between 200 and 1800 m (Herrera-Mejía and Hoyos 2019). As ABL height is influenced by atmospheric stability conditions, dispersion of pollutants could be favoured and hindered under unstable and stable meteorological conditions, respectively (Correa et al. 2009; Rendón et al. 2020). It is in the ultimate case when formation of thermal inversion layer prolongs, increasing the exposure to atmospheric pollutants. Mostly, thermal inversion and high PM concentration episodes take place between 17:00 and 10:00 during March–April and October, when the meteorological conditions are of low-height cloudiness are presented (AMVA 2017a).

Although the increasing concern about inorganic pollutants and carbonaceous content (including organic pollutants such as polycyclic aromatic hydrocarbons (PAHs)) determination in PM, there are few studies focused on polluted and densely populated regions settled in tropical narrow valleys, characterised by weather conditions that hinder atmospheric pollutants’ dilution (Mueller et al. 2019; Zalakeviciute et al. 2020). Moreover, no reports regarding spatio-temporal variations of trace metal(oid)s and PAHs concentrations in Aburrá Valley have been found in literature, whilst PM source apportionment studies are scarce in Latin America, especially in high altitude cities (with low-density air) (Zalakeviciute et al. 2020). The present work aims to assess the chemical composition (encompassing elements and metal(oid)s, PAHs and organic content) of PM_10_ in several sites of Aburrá Valley during 2017, providing novel contribution to the field due to the lack of studies in the area. Also, spatio-temporal variations in the area and PM_10_ sources will be explored, whilst carcinogenic human health risks will be assessed by following the United States Environmental Protection Agency’s (USEPA) guidelines.

## Material and methods

### Study area description

**Fig. 1:**
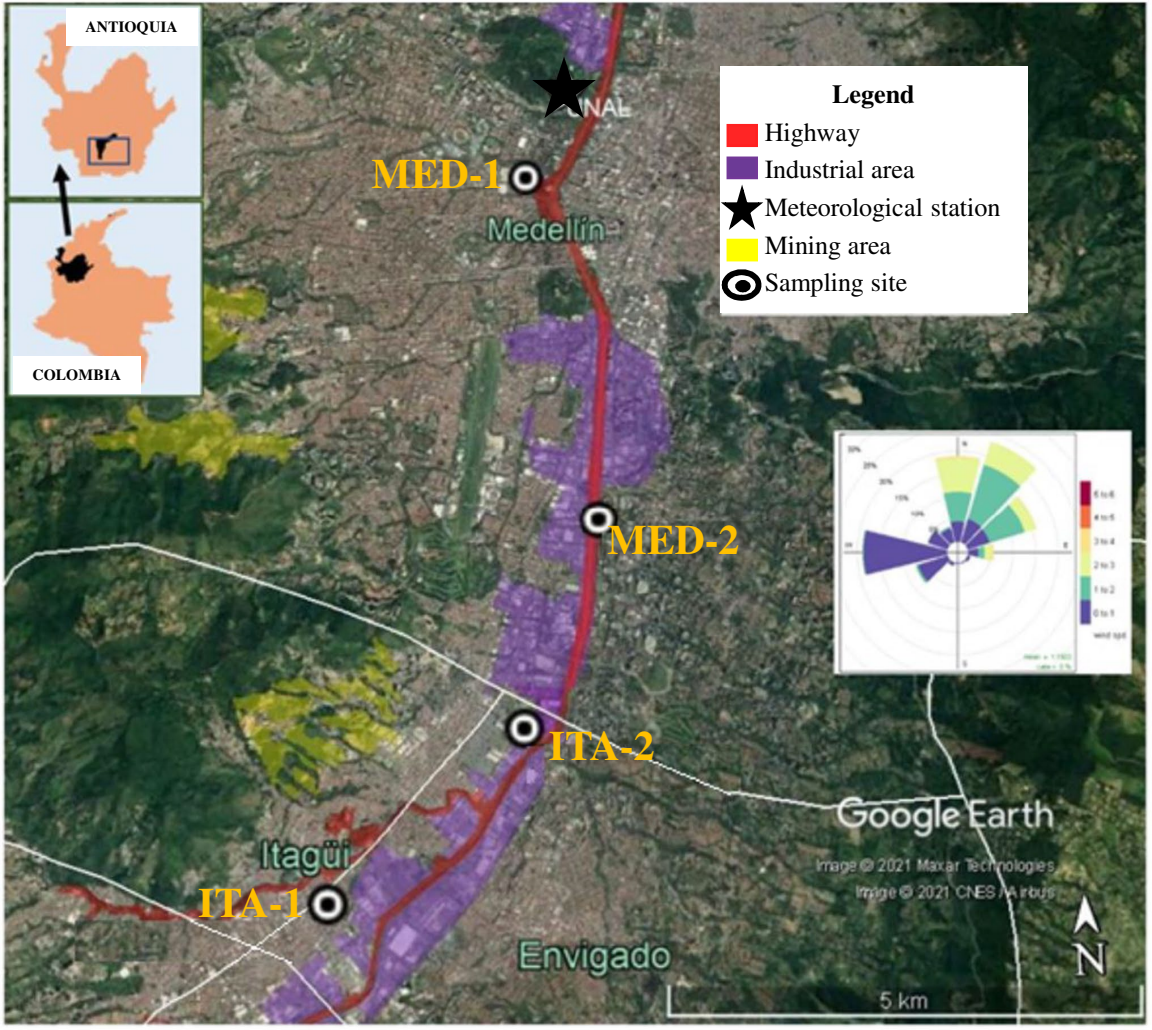
Location of sampling sites at Aburrá Valley South Zone. Source: Google Earth®. Windrose corresponds to average value of speed and direction wind during the study

AMVA is a densely populated region (3.220 inhabitants per square kilometre) with eminently urban characteristics (DANE 2018), where Medellín is the main city (1500 masl). The combination of diverse anthropogenic emission sources (household, industrial and vehicular emissions) with tropical climate and topographical peculiarities, make ANVA one of the most polluted sites in Colombia. In addition, air masses movement of the Intertropical Convergence Zone causes a bimodal cycle of precipitation in the area throughout the year, with a first rainy season (from March to May) and a second one (from September to November) (CICE 2017). In the present study, PM_10_ samples were collected in four sampling stations (belonging to the ANVA Air Monitoring Network) at the south-central area of AMVA, comprising Medellín (MED-1 and MED-2) and Itagüí municipalities (ITA-1 and ITA-2). Further details concerning location of sampling sites are given in Fig. 1 and STable 1 (Supporting Information, SI). Also, meteorological data (wind speed, solar radiation and rainfall) were measured at the station of the air quality network near MED-1. Moreover, data from continuous PM_10_ and PM_2.5_ measurements were acquired from the Air Monitoring Network.

### Sampling and PM_10_ mass determination

PM_10_ samples were collected on quartz-fiber filters (203 × 254 mm, Whatman) for 24 h at 66.6 m^3^ h^−1^ according to US reference method (US Government 1991) by using Graseby-Andersen GBM2360 high-volume samplers (Graseby-Andersen Inc., Smyrna, GA). Quartz-fiber filters were pre-heated at 450 °C for 4 h (for organics contamination removal) before using. After sampling and filter conditioning (by keeping a temperature and humidity of 25 °C and 50%, respectively, for 24 h), PM_10_ mass was gravimetrically determined. Afterwards, filters were stored at –20 °C until further analysis. Field blanks (blank filters placed inside the samplers without PM collection) were also collected along with daily samples and analysed following the same procedure. A total of 104 samples were collected simultaneously from the four sampling sites between March and October 2017.

### Analytical methods for chemical characterisation

#### Metal(oid)s extraction and quantification

Four circular pieces of quartz filters (diameter of 16 mm) were taken from each PM_10_ filter using a steel puncher (Selecta, Barcelona, Spain) and subjected to acid digestion (Piñeiro-Iglesias et al. 2003). In brief, filter portions of each sample were transferred to a polytetrafluoroethylene (PTFE) digestion bomb with 2.5 ml of nitric acid (Baker®, Phillipsburg, PA, USA) and 5 ml of concentrated hydrochloric acid (Baker®) and heated at 90 °C for 12 h. After addition of 2.5 ml of perchloric acid (Baker®) and 1 ml of nitric acid, the mixture was driven to dryness. Finally, residue was reconstituted by adding 2.5 ml of nitric acid and taken to 25 ml using ultrapure water.

Quantification of the isotopes ^27^Al, ^75^As: ^137^Ba, ^9^Be, ^209^Bi,^44^Ca, ^111^Cd, ^59^Co, ^52^Cr, ^133^Cs ^63^Cu, ^56^Fe, ^39^ K, ^7^Li, ^24^ Mg, ^55^Mn, ^95^Mo, ^23^Na, ^60^Ni, ^29^Si, ^31^P, ^208^Pb, ^121^Sb, ^78^Se, ^118^Sn, ^87^Sr, ^205^Tl, ^51^ V and ^66^Zn was performed by inductively coupled plasma mass spectrometry (ICP-MS) (Thermo Finnigan X Series, Waltham, MA, USA) in the peak jump mode under the following instrumental conditions: radio frequency (RF) power 1350 W, nebuliser gas flow 0.8 L min^−1^, auxiliary gas flow 0.9 L min^−1^, and plasma gas flow 15.0 L min^−1^. Calibration graphs were constructed with aqueous standard metal(oid)s solutions (with 2.0 M nitric acid) covering a concentration range of 0 to 2000 μg L^−1^ (STable 2). Also, ^45^Sc, ^72^Ge, ^89^Y, ^103^Rh and ^115^In were used as internal standards. At least one procedural blank (field blanks subjected to the same digestion procedure as PM_10_ samples) was analysed in each extraction batch. The limits of quantification (LOQs) (mean blank ± 10 standard deviation (SD) criterion) were estimated by analysing 11 procedure blanks (STable 2), being in the range of 0.05 (Tl) and 930 ng m^−3^ (Al). In addition, trueness of the method was assessed by analysing the SRM 1649a urban particulate matter reference material (National Institute of Standards and Technology, Gaithersburg, MD, USA) in triplicates. Concentrations found are in good agreement with the certified values (STable 2) after statistical evaluation by applying a t test at 95% confidence level for two degrees of freedom. A statistical summary of metal(oid)s concentrations found in PM_10_ samples (*N* = 104) are shown in STable 3.

#### Stable isotopic δ^13^C quantification

The isotope ratios of δ^13^C in the PM_10_ samples were determined by mass spectrometer of isotopic proportions (Delta V Advantage, Thermo Fisher Scientific, Whaltman, MA, USA) calibrated with certified reference materials (NBS-22, IAEA-CH- 6 and USGS 24) from International Atomic Energy Agency-IAEA (Vienna, Austria), coupled to an elemental sample analyser (Flash EA1112 HT, Thermo Fischer Scientific, Bremen, Germany). Procedures and details for δ^13^C quantification are shown in SI.

#### Equivalent black carbon, total organic carbon and elemental analysis

Equivalent Black Carbon (eBC) were determined by using an optical transmissometer Model OT-21 (Magee Scientific, California, USA), whereas a ThermoQuest Flash EA 1112 (ThermoQuest, Rodano, Italy) elemental analyser were used for the analysis of H, C, S and N and Total Organic Carbon (TOC) (after sample acidification) according to a previous study (Fernández-Amado et al. 2018). Further details concerning eBC quantitation procedure are given in SI, whilst statistical summary of eBC, TOC and elemental (H, C, S and N) concentrations in PM_10_ samples are shown in STable 3.

#### Polycyclic aromatic hydrocarbon extraction and quantification

PAHs were extracted from PM_10_ samples extracted by Pressurised Hot Water Extraction (PHWE) (using an ASE 200 accelerated extraction solvent system, Dionex, Sunnyvale, CA, USA) with water:methanol (3:1) as extracting solvent (Ramos-Contreras et al. 2019). Six circular pieces (16 mm diameter) of each sample were placed in extraction cells with cellulose filters at both ends and spiked with 150 μL of deuterated-labelled PAHs surrogate solution mix (naphthalene-d_8_, acenaphthylene-d_8_, phenanthrene-d_10_, fluoranthene-d_10_, pyrene-d_10_, chrysene-d_12_, benzo[e]pyrene-d_12_ and benzo[g,h,i]perylene d-12 in hexane, 200 μg L^−1^). A single extraction cycle was performed at 200 °C and 2000 psi, with a static time of 5 min. Once finished, extracts were driven to 40 ml with water: methanol (3:1). Subsequently, 15 mL aliquots were pre-concentrated and cleaned up by Membrane Assisted Solvent Extraction (MASE) using a Gerstel device (Mülheim, Germany) consisting of a 20-mL glass vial and a membrane insert made of dense polypropylene (4-cm long with a wall thickness of 0.03 mm and an internal diameter of 6 mm). Membranes were filled with 500 µL of internal standard solution (antracene-d_10_ and dibenzo[a,h]anthracene-d_14_ in hexane, 100 µg L^−1^) and the vial was sealed with a metallic crimp cap provided with PTFE septa. MASE devices were orbitally shaken (730 rpm) and incubated (30 ºC) during 90 min by using a Combi PAL autosampler (CTC-Analytics, Zwingen, Switzerland) tool.

Subsequently, PAHs in hexane extracts were quantitated by a Thermo-Finnigan Trace GC chromatograph (Waltham, MA, USA) equipped with the GC PAL autosampler (CTC-Analytics, Zwingen, Switzerland), Programmed Temperature Vaporizing (PTV) injector and coupled to an ion trap mass spectrometer (Polaris Q), using the Xcalibur software as data processor. A PTV injector provided with a glass wool packed PTV Silcosteel® liner with 2 mm of inner diameter (Thermo Finnigan, Thermo Electron Corporation, Waltham, USA) was used, setting a sample injection volume of 25 µL and a PTV programme starting at 55 °C and heated at 3 °C s^−1^ until 300 °C (held for 20 min). The separation was performed with a DB-XLB column (60 m × 0.25 mm, 0.25 µm film thickness) (J& W Scientific, Folsom, CA, USA), whilst GC oven temperature started at 50 °C (3 min), increased by 4 °C min^−1^ to 325 °C, and held for 20 min. The mass spectrometer [electron impact (EI); 70 eV] operated in tandem mass spectrometry detection mode. Transfer line and ion source temperatures were set at 300 °C and 270 °C, respectively. Helium (99.9999%) was used as the collision gas at the ion trap chamber, and as the carrier gas, under a constant flow rate of 1 mL min^−1^.

A total of 23 PAHs were analysed, comprising naphthalene (NAP), methylnaphthalene (Me-NAP), acenaphthene (ACE), acenaphthylene (ACY), fluorene (FLU), methyl fluorene (Me-FLU), dibenzothiophene (DBT), phenanthrene (PHE), anthracene (ANT), methyl anthracene (Me-ANT), fluoranthene (FLT), pyrene (PYR), retene (RET), benzo[a] anthracene (BaA), triphenylene (TPY), chrysene (CHR), benzo[b + j]fluoranthene (BbjF), benzo[k]fluoranthene (BkF), benzo[e]pyrene (BeP), benzo[a]pyrene (BaP), dibenzo[a,h] anthracene (DahA), indene(1,2,3-c,d)pyrene (IcdP) and bezo[g,h,i] perylene (BghiP). External calibration graphs were carried out in a concentration range of 0 to 300 µg L^−1^ for linearity check, being correlation coefficients (R^2^) between 0.9965 to 0.9994 for all the PAHs. The limits of quantification (LOQs) calculated as mean blank + 10 SD criterion (*N* = 11 procedure blanks) were 0.075, 0.039, 0.005, 0.003, 0.003, 0.021, 0.051, 0.011, 0.015, 0.004, 0.003, 0.003, 0.002, 0.001, 0.001, 0.001, 0.006, 0006, 0.006, 0.002, 0.002, 0.010 and 0.01 ng m^−3^, for NAP, Me-NAP, ACY, ACE, FL, Me-FL, DBT, PHE, ANT, Me-ANT, FLT, PYR, RET, BaA, TPY, CHR, BbjF, BkF, BaP, BeP, DahA, IcdP and BghiP, respectively; being low enough to perform PAHs quantification in studied PM_10_ samples. The inter-day precision and trueness of the analytical procedure were estimated by analysing the SRM 1649b Urban Dust (National Institute of Standards and Technology, Gaithersburg, MD, USA) within different days (*N* = 8). All PAHs demonstrated good inter-day precision, obtaining relative standard deviations (RSDs) of 4.9 to 22.3% for Me-NAP and ACY, respectively; whilst analytical recoveries obtained from SRM 1649b analysis were satisfactory (65 to 117%). A statistical summary of PAHs concentrations found in PM_10_ samples (*N* = 104) are shown in STable 4.

### Source apportionment

Positive Matrix Factorization (PMF) has been used by many researchers to recognise and characterise the major PM_10_ sources. Therefore, a PM apportionment study to estimate the possible contribution of different PM sources in ANVA area was performed by using PMF software provided by USEPA (EPA PMF 5.0 software) (Paatero and Hopke 2003; USEPA 2014; Norris et al. 2014).

### Human health risk assessment of PM_10_-bound metal(oid)s and PAHs

The cancer risk of PM_10_-bound metal(oid)s and PAHs was evaluated according to the USEPA’s human health risk assessment models, being further described in the SI (USEPA 2009).

## Results and discussion

### PM_10_ mass concentration

**Fig. 2:**
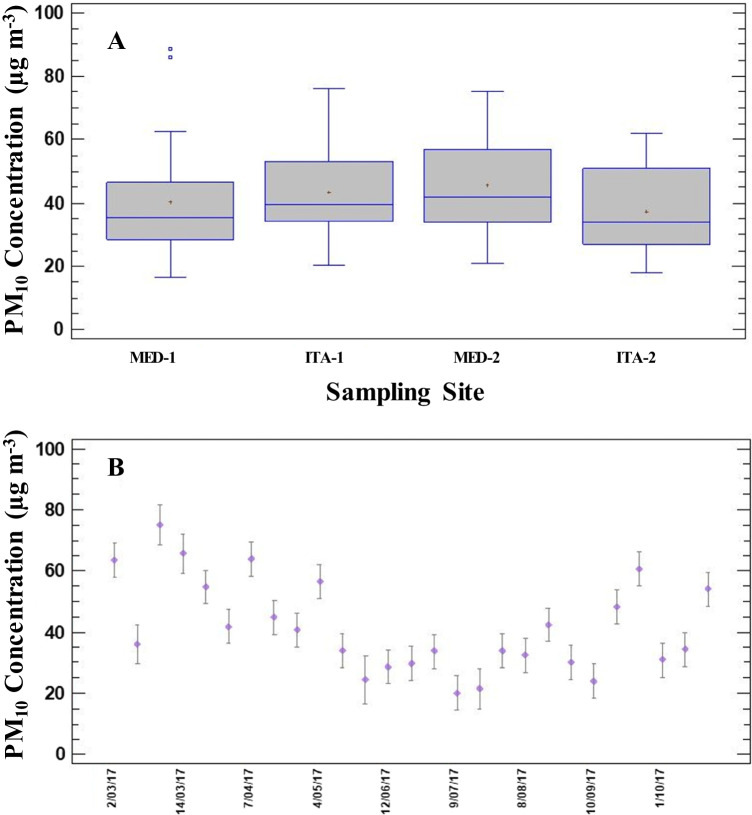
Spatial (**A**) and temporal (**B**) variation of PM_10_ mass (µg m^−3^)

The average PM_10_ concentration in the study area was 41.6 µg m^−3^ (STable 3), ranging from 37.0 µg m^−3^ (ITA-2) to 45.7 µg m^−3^ (MED-2) (Table 1, Fig. 2A). Although PM_10_ mean concentration is below the Colombian annual limit value (50 µg m^−3^) (RC- MADS 2017); it is quite above the limit set by the World Health Organization guidelines (15 µg m^−3^) (WHO 2021). Compared to mean values reported in Bogotá (most populated city in Colombia), average concentration found in the present study is lower than mean concentrations reported for traffic (53 µg m^−3^) or industrial sites (110 µg m^−3^), whereas it is quite similar to mean concentration found in residential areas (41.4 µg m^−3^) (Vargas et al. 2012).

**Table 1:** Mean, minimum (Min) and maximum (Max) values of PM_10_ mass (µg m^−3^) and elements, equivalent black carbon (eBC) and total organic carbon (TOC) concentrations (ng m^−3^) found in each sampling site

	MED-1 (*N* = 27)			MED-2 (*N* = 25)			ITA-1 (*N* = 27)			ITA-2 (*N* = 25)		
	Mean	Min	Max	Mean	Min	Max	Mean	Min	Max	Mean	Min	Max
PM_10_ mass	40.1	16.5	88.7	45.7	21.0	75.3	43.7	22.3	76.0	37.0	18.1	61.8
Al	8575	< 930	51,709	9313	< 930	51,292	8385	< 930	52,922	6712	< 930	45,580
As	1.3	0.31	3.4	1.6	0.34	5.3	2.2	1.0	4.5	1.6	0.24	4.1
Ba	103	< 7.3	578	115	< 7.3	606	95.8	11.7	540	76.6	9.1	485
Be	0.19	< 0.09	0.98	0.23	< 0.09	1.4	0.23	< 0.09	1.3	0.19	< 0.09	1.2
Bi	1.5	< 0.15	5.0	1.8	< 0.15	7.4	1.6	< 0.15	7.4	1.8	< 0.15	7.6
Ca	8580	< 820	41,249	10,506	< 820	84,893	10,048	< 820	83,873	9051	< 820	74,990
Cd	0.47	< 0.17	1.7	0.66	< 0.17	1.6	0.75	0.21	1.4	0.80	< 0.17	2.0
Co	0.73	< 0.19	2.0	0.87	< 0.19	2.7	1.0	0.22	2.8	0.79	< 0.19	2.6
Cr	16.9	< 6.8	94.0	19.1	< 6.8	85.4	16.7	< 6.8	83.9	16.2	< 6.8	73.3
Cs	0.38	< 0.62	1.4	0.36	< 0.62	1.5	0.34	< 0.62	1.5	0.30	< 0.62	1.3
Cu	25.6	< 9.6	72.1	73.9	< 9.6	183	43.6	21.3	89.5	20.9	< 9.6	60.4
Fe	680	212	1518	899	206	1756	616	214	1173	653	224	2834
K	492	< 96.0	1207	992	< 96.0	12,357	962	< 96.0	12,185	960	< 96.0	10,894
Li	4.0	< 5.9	11.0	4.3	< 5.9	17.3	4.2	< 5.9	17.3	3.8	< 5.9	14.5
Mg	6249	< 203	33,189	7038	< 203	46,032	6368	< 203	44,147	5585	< 203	39,890
Mn	14.7	3.6	36.4	19.7	< 3.0	65.5	17.0	4.2	45.3	14.7	3.9	35.0
Mo	12.3	< 17.2	37.1	12.1	< 17.2	40.5	9.6	< 17.2	30.3	12.5	< 17.2	38.9
Na	8066	< 547	14,759	7419	< 547	14,006	7045	< 547	13,523	7438	< 547	13,072
Ni	1.7	< 1.5	6.7	1.9	< 1.5	10.1	2.2	< 1.5	6.8	1.9	< 1.5	7.0
P	341	< 76.2	1552	310	< 76.2	1158	319	< 76.2	1398	329	< 76.2	1467
Pb	17.0	5.6	41.8	16.9	4.5	60.2	13.9	3.7	68.1	17.8	4.6	65.8
Sb	5.6	0.71	19.3	6.2	0.88	13.1	5.2	1.3	13.7	4.9	1.8	10.8
Se	1.9	< 3.7	4.7	2.4	< 3.7	6.1	2.7	3.7	6.1	2.1	3.7	4.5
Sn	8.3	< 3.1	35.5	13.2	< 3.1	34.9	11.0	< 3.1	34.9	10.6	< 3.1	28.5
Sr	23.9	< 2.1	140	25.6	< 2.1	143	24.0	< 2.1	136	20.2	< 2.1	133
Tl	0.07	< 0.05	0.27	0.11	< 0.05	0.22	0.16	< 0.05	0.56	0.25	< 0.05	0.68
V	2.1	0.36	5.6	2.3	0.47	5.4	3.3	0.96	7.7	2.1	0.54	4.3
Zn	86.0	< 20.8	299	129	< 20.8	426	140	27.1	422	211	34.4	805
C	8597	3310	16,300	10,250	3280	16,420	8762	4050	14,060	7743	3970	13,560
H	1408	410	12,260	1559	270	15,090	1388	410	11,830	1405	100	14,730
N	617	310	1090	696	270	1450	696	340	1390	593	310	1150
S	737	< 40.0	3100	535	< 40.0	1480	917	< 40.0	3270	950	< 40.0	3670
eBC	3235	1206	5447	3746	1177	5245	3168	1573	4720	2831	1306	4605
TOC	8925	3390	15,670	10,630	3220	16,020	9177	4110	15,250	8180	4070	14,220

As can be seen from SFigure 1, mean daily fluctuation of PM_10_ concentration shows two peaks at 08:00–10:00 and 18:00–20:00, which seems to be associated with the peak working hours when anthropogenic activity is expected to be higher (mainly vehicular traffic sources). However, a thorough study considering the climate should be considered as it may affect PM_10_ levels significantly (Herrera-Mejía and Hoyos 2019; Roldán-Henao et al. 2020). Additionally, PM_10_ levels observed for the sampling sites (MED-1, MED-2, ITA-1 and ITA-2) showed no statistically significant differences (*p* > 0.05) among them (Fig. 2B). Regarding temporal fluctuations, maximum PM_10_ levels were reported during March (Fig. 2B), which might be resulted from high cloudiness and thermal inversion episodes that mostly occur in the transition to the rainy season (SFigure 2, in SI).

### Elemental composition of PM_10_ and source apportionment

Elements concentrations of PM_10_ collected at the four sampling sites (MED-1, MED-2, ITA-1, ITA-2), together with eBC and TOC concentrations, are summarised in Table 1. Significant positive correlations were observed between PM_10_ mass and eBC, TOC and C concentrations (r > 85%), which could point to a common source. On this background, carbon content accounted for 32.1% and 32.9% (for ITA-1 and ITA-2, respectively) of the PM_10_ material in all studied areas (Table 2). Similar results were reported in Kennedy site (Bogotá, Colombia), where the PM_10_ carbon content was associated to contribution of both medium and small industries and road traffic sources (Vargas et al. 2012).

**Table 2:** PM_10_ sources identified for each site, along with their associated main constituents

Site	Identified factors	Contribution	
		(%)	Main constituents
MED-1	1. Re-suspended powder	7.5	Na, Mg, Al, Ca, Sr, Ba, Bi
	2. Recovery of batteries and other nonferrous smelting	9.9	Pb
	3. Combustion	32.5	H, C, TOC, eBC
	4. Quarries (rocks and clays)	21.1	Cr, V, Mn, Fe, Co, Sb, K
	5. Secondary aerosols/ions	23.3	S, N
	6. Tire wear	12.7	Zn, As, Cd
MED-2	1. Re-suspended powder	6.8	Na, Mg, Al, Ca, Sr, Ba
	2. Quarries (rocks and clays)	19.8	Cr, Fe, Mn, Co
	3. Combustion, mobile sources	32.8	eBC, C, TOC, N
	4. Secondary aerosols/sulphates	14.6	S
	5. Recovery of batteries and other no ferrous smelting	10.9	Pb, Cu
	6. Tire wear	15.0	Ni, Zn, As, Sn
ITA-1	1. Recovery of batteries and other no ferrous smelting	7.8	H, Pb
	2. Tire wear	9.6	Ni, Zn
	3. Re-suspended powder	7.4	Na, Mg, Al, Sr, Ba, Bi
	4. Secondary aerosols/sulphates	13.2	S
	5. Combustion, mobile sources	32.1	eBC, TOC, C, N
	6. Quarries (rocks and clays)	29.9	Cr, V, Fe, Mn, Co, Cd
ITA-2	1. Re-suspended powder	2.2	Na, Mg, Al, Ca, Sr, Ba
	2. Recovery of batteries and other no ferrous smelting	16.6	Pb, Ni
	3. Combustion	32.9	eBC, TOC, C, N
	4. Secondary aerosols/sulphates	14.8	S
	5. Tire wear	2.5	Zn, Tl
	6. Quarries (rocks and clays)	31.1	Cr, V, Mn, Fe, Co, Cd, Sn

Among the elements, the highest concentrations were observed for Al, Ca, Mg and Na, which could be associated to a common PM crustal origin (high correlation observed for them (*r* > 86%, SFigure 3), being mostly derived from soil and dust resuspension (Khodeir et al. 2012; Zalakeviciute et al. 2020), whilst metals such as Li, Be and Cs were not quantitated in most samples (STable 3, in SI). Moreover, concentrations of carcinogenic metal(oid)s in PM_10_ ranged between 0.24–5.3 ng m^−3^ (As), < 0.17–2.0 ng m^−3^ (Cd), < 0.19–2.8 ng m^−3^(Co), < 1.5–10.1 ng m^−3^(Ni) and 3.7–68.1 ng m^−3^ (Pb) (Table 1), being all their averages below the annual values set by the Colombian government (5.0, 180 and 500 ng m^−3^ for Cd, Ni and Pb, respectively) (RC-MADS 2017). Despite scarce studies concerning PM chemical characterisation in tropical sites have been reported, metal(oid)s levels found is this research were compared to studies conducted in other tropical areas. Results obtained were lower than those recently reported at Quito city (Ecuador) by Zalakeviciute et al. (Zalakeviciute et al. 2020) and similar to those reported in Bogotá city (Colombia) (Ramírez et al. 2018; Vargas et al. 2012).

As commented above, PMF model 5.0 was applied to estimate possible PM sources in the area. Exploratory tests were performed to set a factor number that would allow an acceptable confidence level (lower Q/Qexp) and identifying the different sources to be separated. For each trial, 200 runs were performed considering a randomly generated seed value. As is illustrated by SFigure 4, stabilisation of the residuals is mostly achieved by setting 6 factors, which were associated to different sources basing on PM elemental proportion (Reff et al. 2007). Results obtained are described in Table 2, whilst source profiles are shown in SFigure 5 (A–D), in SI. The fitness between PMF model and PM_10_ data was mainly successful (*r*^2^ values between 0.93 and 0.97), whilst those elements which did not show a good fit (*r*^2^ < 0.5) were excluded from the model. Elements were categorised according to their signal to noise (S/N) ratio (Brown et al. 2015; Paatero and Hopke 2003), considering strong variables those elements that showed a S/N ratio greater than or equal to five, whilst those elements whose S/N ratio was under detection limit was sorted as weak variables (STable 6, in SI).

**Fig. 3:**
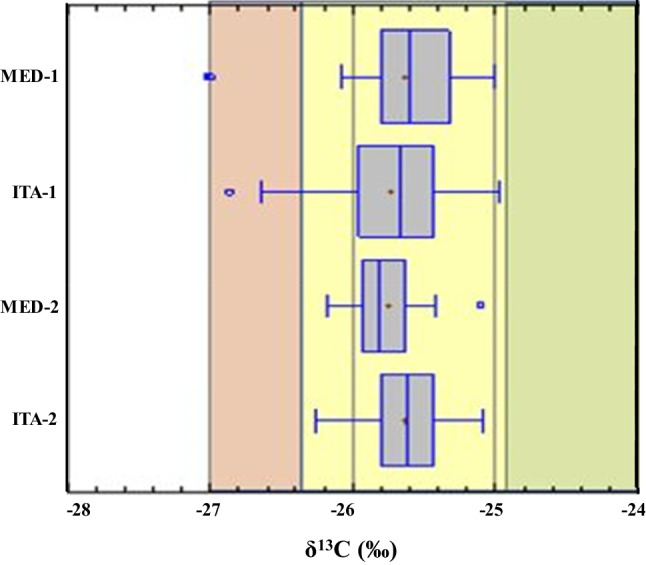
Fuel source association for each sampling site, basing on δ^13^C (‰) analysis.  : (regular fuel),  : diesel (Cao et al. 2011; Widory 2006)

The contribution to combustion processes source was similar in all sampling sites (around 32%), being mainly attributed to vehicle traffic sources due to the high eBC, C and TOC contents. Then, all sites are categorised could be defined as urban, with a certain traffic and industrial activity influence. On this basis, the organic PM fraction could be attributed to smaller particles such as PM_2.5_, corresponding to approximately 58% of PM_10_ (SFigures 1 and 6, in SI), which agrees with studies conducted at urban industrial areas (Khodeir et al. 2012; Spandana et al. 2021; Sugimoto et al. 2016). Also, some metal(oid)s are frequently associated to anthropogenic PM sources such as road dust tracers (Al, Mn, K and Sr), fuel oil combustion (V) and burning waste or abrasive wear of tires (Zn) (Fauser et al. 2002); construction activities (Cr) (Watson and Chow 2015); and smelting of non-ferrous material, battery recycling and waste incineration (Pb) (Landis et al. 2017). As the use of Pb as an antiknock agent in gasoline has been banned in Colombia since 1991, Pb may be released to atmosphere as a result of local anthropogenic sources. Since Pb concentrations are not correlated with other elements, the highest concentrations of Pb observed in ITA-1 and ITA-2 sites (13.9 ng m^−3^ and 17.8 ng m^−3^, respectively) could be associated with winds from the south and southwest (1.4–1.6 m s^−1^), where industrial activity is significant. The great contribution of Co, Cr, Fe, K, Mn, Sb and V (linked to extraction of materials and stone) in ITA-1 and ITA-2 sites could be due to the proximity to quarries in operation located at municipality of Itagüí (Table 2). Sources attributed to secondary aerosols offered a less contribution (13.2 to 23.3%), with respect to sources associated with combustion processes (32.1 to 32.9%) (Table 2).δ^13^C values were between − 25.0 and − 27.0‰ (STable 7, in SI), which support the predominant traffic emission in all the sites (Cao et al. 2011; Widory 2006). Results observed were similar to those reported at places such as Mexico City (δ^13^C: − 26.3 to − 24.3‰) (López-Veneroni 2009), Paris (δ^13^C: − 26.75 to − 25.75‰) and Tuscany (δ^13^C: − 26.5 to − 25.5‰) (Grassi et al. 2007). As is shown by Fig. 3, δ^13^C values found seem to be related to gasoline and diesel emissions rather than carbon emissions since values in commercial diesel samples were reported to be between − 33.3 to − 25.8‰ (Muhammad et al. 2015). In addition, results observed are in agreement with reports regarding consumption of diesel (about 110 mill gallons) and gasoline (about 190 mill gallons) during 2016 in AMVA (AMVA 2017b)*.*

Stationary sources associated to the use of coal are not predominant in AMVA, accounting for 19% of PM_2.5_ emissions. In this regard, PM_10_ emissions from stationary sources were mainly associated with three industrial sectors: textile, beverage, food and tobacco and chemical (SIGAIRE 2022).

### PAHs analysis in PM_10_

**Fig. 4:**
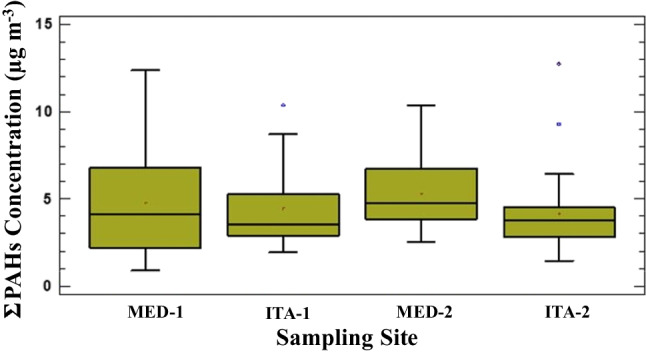
Spatial variation of PAHs levels (ng m^−3^)

PAHs concentrations (mean, median, minimum, maximum and SD) in PM_10_ samples in each sites during the whole period are given in Table 3. The Kruskal–Wallis analysis showed no statistically significant differences (*p* < 0.05) between PAHs concentrations found in each sampling site (Fig. 4). High molecular weight PAHs (5 and 6 condensed rings) were predominant in all samples collected (Table 3 and SFigure 7, in SI), being BghiP, BbjF and IcdP the compounds that showed the highest concentration values (between 0.093–1.6 ng m^−3^, 0.069–2.7 ng m^−3^ and < 0.001–1.6 ng m^−3^ for BghiP, BbjF and IcdP, respectively) (Table 3).

**Table 3:** Mean, minimum (Min) and maximum (Max) values of individual PM_10_-bound PAHs, PAHs summation (ΣPAHs) and equivalent BaP (BaP_eq_) concentrations (ng m^−3^) found in each sampling site

	MED-1			MED-2			ITA-1			ITA-2		
	Mean	Mín	Max	Mean	Min	Max	Mean	Min	Max	Mean	Min	Max
NAP	0.17	< 0.075	0.36	0.29	< 0.075	2.0	0.20	< 0.075	1.0	0.18	< 0.075	1.0
Me-NAP	0.22	< 0.039	0.68	0.43	0.082	3.4	0.26	0.088	1.6	0.68	0.040	7.5
ACE	0.035	< 0.003	0.10	0.052	0.013	0.34	0.026	< 0.003	0.072	0.019	< 0.003	0.042
ACY	0.011	< 0.005	0.076	0.032	< 0.005	0.30	0.017	< 0.005	0.083	0.015	< 0.005	0.093
FLU	0.037	0.010	0.12	0.083	0.006	0.69	0.055	0.012	0.16	0.045	< 0.003	0.19
Me-FLU	0.024	< 0.021	0.22	0.17	< 0.021	1.2	0.051	< 0.021	0.48	0.098	< 0.021	0.52
PHE	0.17	0.050	0.44	0.23	0.089	0.61	0.17	0.074	0.34	0.12	0.019	0.27
ANT	0.051	< 0.015	0.14	0.075	< 0.015	0.38	0.056	< 0.015	0.14	0.036	< 0.015	0.078
Me-ANT	0.016	< 0.004	0.082	0.026	< 0.004	0.13	0.018	0.005	0.065	0.011	< 0.004	0.045
FLT	0.14	0.019	0.41	0.14	0.071	0.22	0.14	0.025	0.35	0.097	0.022	0.24
PYR	0.22	0.027	0.59	0.20	0.098	0.33	0.19	0.054	0.45	0.13	0.036	0.33
RET	0.013	< 0.002	0.14	0.005	< 0.002	0.072	0.041	< 0.002	0.29	0.005	< 0.002	0.072
BaA	0.098	< 0.001	0.37	0.078	< 0.001	0.22	0.077	< 0.001	0.25	0.028	< 0.001	0.13
TPY	0.10	0.033	0.24	0.086	< 0.001	0.18	0.099	0.015	0.33	0.064	< 0.001	0.14
CHR	0.22	0.021	0.58	0.18	0.033	0.35	0.19	0.057	0.52	0.13	0.034	0.27
BbjF	0.85	0.069	2.7	0.74	0.18	1.3	0.78	0.20	2.3	0.60	0.13	1.3
BkF	0.21	0.016	0.74	0.17	0.058	0.27	0.22	0.051	0.85	0.16	0.040	0.31
BeP	0.18	0.026	0.66	0.14	0.041	0.25	0.17	0.037	0.76	0.094	0.011	0.20
BaP	0.32	0.040	0.95	0.27	0.082	0.46	0.28	0.082	0.78	0.25	0.067	0.54
DahA	0.059	< 0.002	0.33	0.016	< 0.002	0.064	0.094	< 0.002	0.52	0.013	< 0.002	0.15
IcdP	0.49	< 0.010	1.2	0.58	< 0.010	1.6	0.54	< 0.010	1.3	0.47	< 0.010	0.93
BghiP	0.86	0.093	1.6	0.82	0.26	1.4	0.84	0.46	1.5	0.82	0.15	1.5
ΣPAHs	4.5	0.900	12.4	4.8	2.5	10.4	4.5	2.0	10.4	4.1	1.4	12.8
BaP_eq_	0.90	0.15	4.9	0.76	0.29	3.3	1.2	0.15	6.1	0.64	0.10	3.8

The sum of PAH concentrations (ΣPAHs) considering all sites ranged from 0.90 to 12.8 ng m^−3^, whilst mean ΣPAHs found in each site ranged between 4.1 (ITA-2) to 4.8 (MED-2) ng m^−3^ (Table 3). Previous studies in Colombia reported values among 1.49–8.55 ng m^−3^ (Σ_16_PAHs, from an exploratory study in AMVA) (Mueller et al. 2019). Also, ΣPAHs found in the present study were higher than those reported in European cities (Callén et al. 2011; Oliveira et al. 2017), but lower than those observed in China (Yin and Xu 2018).

Concerning PAHs contribution, low ring-number (2–3 rings) PAHs (Σ_2–3rings_PAHs: NAP, Me-NAP, ACE, ACY, RET, FLU, Me-FLU, PHE, ANT and Me-ANT) accounted for 16–30% of ΣPAHs probably due to their volatility (being mainly part of atmospheric gaseous phase), whereas some PAHs such as DBT and other volatile PAHs (Me-FLU, ACY and RET) were found in concentrations < LOQs in most of PM_10_ samples (STable 4, in SI). Middle ring number (4 rings) PAHs (Σ_4rings_PAHs: FLT, PYR, BaA, TPY and CHR) accounted for 10–15%, whilst high ring-number molecules (Σ_5–6rings_PAH), BeP, BbjF, BkF, BaP, IcdP, DBahA and BghiP were predominant in all sampling sites (accounting for 59–67%). Several studies associated emissions of low molecular weight PAHs (≤ 4 rings) to diesel and heavy vehicles; being release of high molecular weight PAHs (5 and 6 rings) linked to emissions from light vehicles or gasoline engines, and considered the PAHs fraction that triggers the most adverse human health effects (Hwang et al. 2003; Liu et al. 2015). Also, the high levels found for 5–6 rings PAHs would support the predominant road traffic source observed in sampling sites.

Carcinogenic PAHs (BbF, CHR, IcdP, BaP, BkF, BaA, DahA) concentrations (Σ_c_PAH) ranged between 1.8 ng m^−3^ (ITA-2) and 1.2 ng m^−3^ (ITA-1), representing 27–44% of the total PAHs levels. Although statistically significant spatial variations of Σ_c_PAH concentrations were not found, monthly differences were observed; observing a low Σ_c_PAH concentration during July (1.0 ng m^−3^), which could be due to an increasing dispersion of pollutants by local and mesoscale meteorological events during this period, as well as due to a minor vehicular traffic during mid-year holidays. Significant correlation (*r* > 70%) was observed between some heavy PAHs (BghiP, BbjF, BkF, CHR) and eBC levels, which might be attributed to a strong adsorption affinity of PAHs in carbon particles (eBC) (Guo et al. 2021).

**Fig. 5:**
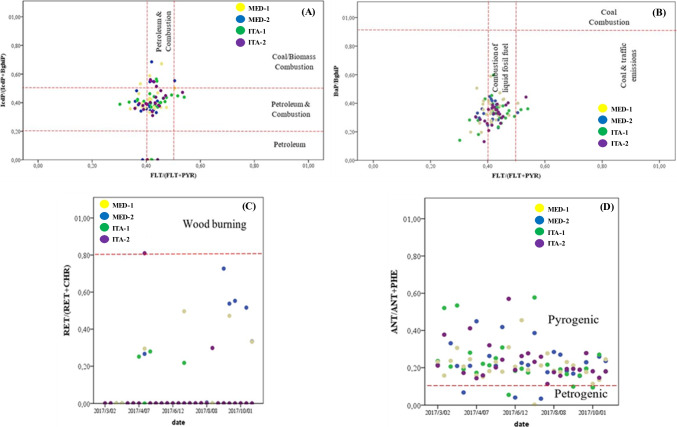
Cross-plots for selected PAHs diagnostic ratios, considering each sampling site: **A** FLT/(FLT + PYR) ratio vs. IcdP/(IcdP + BghiP) ratio, **B** FLT/(FLT + PYR) ratio vs. BaP/BghiP ratio, **C** Date vs. RET/(RET + CHR) ratio, and **D** Date vs. ANT/(ANT + PHE) ratio (Park et al. 2011; Tobiszewski and Namieśnik 2012)

Analysis of molecular diagnostic ratios suggested that around 70% of the emissions of PM_10_-bound PAHs would be associated with combustion of liquid fuel (0.4 < FLT/(FLT + PYR < 0.5) and around 9% would be linked to biomass or diesel combustion (IcdP/IcdP + BghiP = 0.37) (Fig. 5A–B); being barely 1% associated to wood burning (RET/RET + CHR > 0.8) (Fig. 4C) and observing no association to coal combustion source (BaP/BghiP > 0.9) (Fig. 5B). Finally, 92% of the emissions of PM_10_-bound PAHs would be linked to pyrogenic processes (ANT/ANT + PHE > 0.1) (Fig. 5D) (Park et al. 2011; Tobiszewski and Namieśnik 2012) (Fig. 5A–D). These results are in accordance with ^13^C data and the AMVA’s emissions inventory for 2015 (AMVA 2017b), reporting that 79.8% of the circulating fleet used gasoline, 15.9% diesel and 3.8% natural gas; and being Medellin the municipality with the highest diesel fuel consumption (over 60 mill gallons/year in 2016) (AMVA 2017b). Therefore, mobile sources were estimated to be about 70% of the contribution to fine particulate matter (AMVA 2017b).

### Health risk assessment

Carcinogenic risks of 1.0 × 10^–6^ and 1.0 × 10^–4^ were set by USEPA to define human carcinogenic health risk thresholds (Davie-Martin et al. 2017; Sah et al. 2017; USEPA 2019): values higher than 1.0 × 10^–4^ would suggest significant carcinogenic adverse risks, whilst carcinogenic risks under 1.0 × 10^–6^ would suggest no significant carcinogenic risk.

### Life-time cancer risk for inhalation exposure to metal(oid)s

Estimated average life-time cancer risks (LCRs) were between 2.8 × 10^–6^ (MED-1) to 4.2 × 10^–6^ (ITA-1) (STable 8, in SI), being As and Co the metal(oid)s that mainly contributed to LCRs (SFigure 8A, in SI), suggesting that the risk would be associated mainly to industrial and mining activities rather than combustion processes. As can be seen from the table, LCRs exceeded the limit of 1.0 × 10^–6^ at several sites. Furthermore, no significant spatial variations were observed for LCRs (*p* > 0.05); however, LCRs estimated in June and July were lower (SFigure 8A–B, in SI). As commented above, this could be explained by the wind speed and temperature variation during those months, favouring pollutants dispersion in the area (SFigure 9–10, in SI). Maximum average wind speed values occur during the month of July, conditions that favour dilution of the pollutants emitted (SFigure 9, in SI). Also, the low average temperatures during March and October slow down the atmospheric dynamics causing a longer exposure to atmospheric pollutants (SFigure 10, in SI).

### Life-time cancer risk (LCR) for inhalation exposure to PAHs

PAHs concentrations were used to estimate the BaP equivalent concentrations (BaP_eq_), using the toxicity factors defined in STable 5 (in SI). The BaP_eq_ calculated ranged between 0.10 and 6.1 ng m^−3^ (Table 3). Several exceedances of the BaP_eq_ (BaP_eq_ concentrations > 1.0 ng m^−3^ set by current Colombian legislation) were observed during the sampling period at all sampling sites (RC-MADS 2017) and the European Commission Guideline for Air Quality (EU 2004). Average LCRs for each sampling site were 9.9 × 10^–7^, 8.4 × 10^–7^, 1.4 × 10^–6^ and 7.0 × 10^–7^ at MED-1, MED-2, ITA-1 and ITA-2, respectively, whilst an average LCR value of 9.8 × 10^–7^ (Table 4) was estimated considering all the sites, suggesting that approximately 1 out of every 1,000,000 people could eventually develop cancer due to inhalation PM_10_-associated PAHs exposure. Although some exceedances of the acceptable risk limit of 1.0 × 10^–6^ (Table 4) were observed at several sites, the average risk estimated during the sampling period for all sites was smaller than the upper risk limit of 1.0 × 10^−4^ set by USEPA (Davie-Martin et al. 2017; USEPA 2019). Also, no statistically significant differences of LCRs between each site was observed (*p* < 0.05). Nevertheless, high risks were estimated during March and May, which could be attributed to an unfavoured dispersion of pollutants due to meteorological events.

**Table 4:** Lifetime cancer risks (LCRs) estimated for PM_10_-bound PAHs exposure via inhalation

Site	*n*	Mean	RSD%	Min	Max
MED-1	27	9.9 × 10^–07^	1.1	1.6 × 10^–07^	5.4 × 10^–06^
MED-2	25	8.4 × 10^–07^	0.82	3.2 × 10^–07^	3.7 × 10^–06^
ITA-1	27	1.4 × 10^–06^	1.4	1.6 × 10^–07^	6.7 × 10^–06^
ITA-2	25	7.0 × 10^–07^	1.2	1.1 × 10^–07^	4.2 × 10^–06^
Total	104	9.8 × 10^–07^	1.3	1.1 × 10^–07^	6.7 × 10^–06^

## Conclusions

In the present study, chemical composition (comprising elements, PAHs, eBC, TOC and δ^13^C carbon isotope ratios) were assessed in a total of 104 PM_10_ samples collected from four sites located at a tropical narrow valley, where pollutants dispersion could be hindered by several meteorological events. Spatio-temporal variability in the area was studied, founding non-significant differences between the concentrations of pollutants within the different sampling sites, which would point that the atmospheric dynamics in the Aburrá Valley, at least in the southern zone, behaves like an atmospheric airshed. Concerning temporal fluctuations, pollutions peaks might be associated with rainy periods, observing the highest concentrations in March–May and lower values in July. Intraday or hourly studies would be recommended to evaluate the effect of valley shape and the influence of daily heating and cooling over metal(oid)s and PAHs concentrations. Besides, PMF results pointed that combustion and mining activities (quarries) were the main PM_10_ source in the studied area. Mean concentration of toxic metal(oid)s (with potential risk even at low concentrations) such as As, Cd, Co, Ni, Pb and Sb were found below the values set by the Colombian regulation, whereas average BaP concentration (0.25–0.32 ng m^−3^) did not exceed the limit value set by directive 2004/107/EC. Among PAHs, BbjF and BghiP were the most profuse in PM_10_ samples, following by IcdP, BaP, BkF, Chry PYR and Me-NAP. Furthermore, 5-ring and 6-ring PAHs accounted for 59–67% of PAHs content, whilst 27–44% of total PAHs concentration was attributed to carcinogenic PAHs. Diagnostic ratios suggested a pyrogenic origin for PM_10_-associated PAHs in all sampling sites. Carcinogenic risks estimated for PM_10_-bound PAHs exposure via inhalation could be considered as moderate, whereas significant inhalation carcinogenic risk was observed for carcinogenic metal(oid)s exposure in the Aburrá valley during the sampling period.

On the basis of the results obtained, the present study would provide useful data to achieve a better understanding of PM_10_ exposure in valleys with limited atmospheric diffusion, where scarce information is available, as well as its health impact on people living in such areas. Also, further studies in the area would be of great interest so as to support suitable policies to decrease population’s exposure.

## Supplementary Information

Below is the link to the electronic supplementary material.

Supplementary file1 (DOCX 1256 KB)

## Data Availability

The datasets generated during and/or analysed during the current study are not publicly available but are available from the corresponding author on reasonable request.
