# Supplementary material for: Source apportionment of PM10 and health risk assessment related in a narrow tropical valley. Study case: Metropolitan area of Aburrá Valley (Colombia)
Source: Environ Sci Pollut Res Int. 2023 Apr 5;30(21):60036–49. doi: 10.1007/s11356-023-26710-1 (PMC10163095; doi:10.1007/s11356-023-26710-1)
Supplement: Supplementary file 1 — Supplementary file1 (DOCX 1256 KB) [file 11356_2023_26710_MOESM1_ESM.docx]

**Source apportionment of PM_10_ and health risk assessment related in a narrow tropical valley. Study case: Metropolitan area of Aburrá Valley (Colombia)**

Carlos Ramos-Contreras^a,b^, María Piñeiro-Iglesias^a^, Estefanía Concha-Graña^a^, Joel Sánchez-Piñero^a^, Jorge Moreda-Piñeiro^a,*^, Amaya Franco-Uría^c^, Purificación López-Mahía^a^, Francisco Molina-Pérez^b^, Soledad Muniategui-Lorenzo^a^.

^a^University of A Coruña. Grupo Química Analítica Aplicada (QANAP), University Institute of Research in Environmental Studies (IUMA), Department of Chemistry. Faculty of Sciences. Campus de A Coruña, s/n. 15071 – A Coruña. Spain.

^b^Grupo de Investigación en Gestión y Modelación Ambiental (GAIA), Escuela Ambiental, Facultad de Ingeniería, Universidad de Antioquia UdeA, Calle 70 No. 52-21, Medellín, Colombia.

^c^Dept. of Chemical Engineering, School of Engineering, University of Santiago de Compostela, 15782 Santiago de Compostela, Spain

^*^Corresponding author. E-mail address: [jorge.moreda@udc.es](mailto:jorge.moreda@udc.es) (Jorge Moreda-Piñeiro)

**Stable Isotopic δ^13^C Quantification**

Two small discs (0.9 cm diameter) placed in a capsule were burned inside an oxidation oven at 1020 ° C in excess of oxygen. The gas produced is transferred to a reduction oven (650 ° C) and separated by gas chromatography. The isotopic ratio in the samples is calculated by the expression:

$\delta^{13}C_{sample}\left( {\%}_{0} \right)=\left[ \frac{\left( {{}^{13}C}/{{}^{12}C} \right)_{sample}}{\left( {{}^{13}C}/{{}^{12}C} \right)_{standard}}-1 \right]\times{10}^{3}$ (1)

The determination of the stable carbon isotope composition in PM_10_ samples could be used for source apportionment study since the ^13^C/^12^C ratio is strongly dependent on the origin of the carbonaceous aerosols (Palma et al. 2018).

**Equivalent black carbon Quantification**

PM_10_ samples together with blank filters were placed in the OT-21 transmissometer (Maegee Scientific) for equivalent black carbon (eBC) measurement. The instrument compares the intensity of the light (l = 880 nm) transmitted through the sample with the intensity transmitted through the reference filter target, defining attenuation (ATN) as follows:

$ATN=100\times ln\left( \frac{I_{0}}{I} \right)$ (2)

where $I$ and $I_{0}$ are the intensities transmitted through the sample and target filters respectively.

Subsequently, ATN can be easily converted to eBC concentration (expressed in μg cm^-2^) according to the following equation:

$ATN=eBC\times\sigma$ (3)

Where σ is the specific attenuation coefficient, being 16.6 cm^2^ μg^-1^ the recommended value for samples collected on quartz fiber filters (Mu et al. 2015).

**Human health risk assessment of PM_10_‑bound metal(oid)s and PAHs**

The carcinogenic risk of PM_10_-bound metal(oid)s was estimated according to the USEPA human health risk assessment models (USEPA 2009). Exposure concentrations (ECs) were estimated for each sample as follows:

$EC=\frac{C\times ET\times ED\times EF}{{AT}_{n}}$ (1)

where C is metal(oid) concentrations in PM_10_ (µg m^-3^), EF is exposure frequency (180 days/year); ED is exposure duration (24 years for adults); ET is exposure time (hours/day), which was 24 h/day in this study and AT_n_ is average time (AT_n_=70 year$\times$365 days/year$\times$24 h for carcinogens). Then, lifetime cancer risks (LCRs) were calculated for each sample by using the equation below:

$LCR=EC\times IUR$ (2)

where IUR (µg m^-3^)^-1^ is the inhalation unit risk for each metal(oid): Cd (1.8 x 10^-3^), Pb (1.2 x 10^-5^), As (4.3 x 10^-3^), Ni (2.6 x 10^-4^) and Co (9.0 x10^-3^), obtained from the USEPA database (USEPA 2009).

In order to estimate carcinogenic risk of PM_10_-bound PAHs, USEPA’s ﻿benzo(a)pyrene-equivalent (BaP_eq_) toxicity approach was considered (USEPA 2009), using toxicity equivalent factors (TEF) for each PAH (shown in STable 5). For those isomers which no TEFs were reported, the values of their most similar congener were used (Samburova et al. 2017). Then, benzo[a]pyrene (BaP) equivalent concentrations (BaP_eq_) were calculated for each sample according to the following equation:

$\left[ {BaP}_{eq} \right]=\sum\left( C_{i}\times{TEF}_{i} \right)$ (3)

where C_i_ and TEF_i_ corresponds to the concentration of the i^th^ PAH and TEF_i_ at its respective relative power factor. The risk of permanent (70 year) exposure to environmental concentrations of certain PAHs (Life-time Cancer Risk) is calculated by the expression:

$LCR=\left[ {BaP}_{eq} \right]*{UR}_{aj}$ (4)

where ${UR}_{aj}$ corresponds to the previously reported risk of cancer from exposure to BaP by inhalation (UR = 1.1 x 10^-6^ (ng / m^3^) ^-1^) age-adjusted , taking into account that the effect of exposure is greater in the early years (Murray and Penning 2018).

**STable 1.** Sampling sites longitude, latitude, altitude; and station class data.

| **ID** | **Site** | **Municipality** | **Latitude (N)** | **Longitude**  **(W)** | **Altitude** | **Class** |
| --- | --- | --- | --- | --- | --- | --- |
| MED-1 | Corantioquia (Environmental Agency) | Medellín | 6.2525° | -75.5861° | 15 m | Mesoscale trend Urban |
| MED-2 | Politécnico Jaime Isaza Cadavid (Educative Institution) | Medellín | 6.2090° | -75.5778° | 4 m | Traffic Urban |
| ITA-1 | School El Rosario F. San Vicente | Itagüí | 6.1745° | -75.6103° | 5 m | Industrial Urban |
| ITA-2 | Wastewater treatment Plant | Itagüí | 6.1745° | -75.6103° | 10 m | Mesoscale trend Urban |

**STable 2.** Figures of merits for elemental analysis by ICP-MS.

| **Element** | **Internal Standard** | **Equation** | **Range**  **(µg L^-1^)** | **r^2^** | **Quantification limit (QL)**  **(ng m^-3^)** | **Reference material 1648a NIST** | | | |
| --- | --- | --- | --- | --- | --- | --- | --- | --- | --- |
|  |  |  |  |  |  | **Obtained Value Mean (N=3)**  **(mg kg^-1^)** | **Reference value**  **(mg kg^-1^)** | **%R** | **RSD** |
| Al | ^45^Sc | y= 0.1609X + 0.154 | QL-2000 | 0.9994 | 930 | 35127 | 34300 ± 1300 | 109 | 7.0 |
| As | ^72^Ge | y= 0.0372X + 0.123 | QL-2000 | 0.9999 | 0.23 | 108 | 115.5 ± 3.9 | 93.9 | 1.5 |
| Ba | ^115^In | y=0.0072X + 0.0771 | QL-2000 | 0.9976 | 7.3 | 734 | 737 | 99.4 | 0.49 |
| Be | ^45^Sc | y=0.0005X + 0.0035 | QL-2000 | 0.9996 | 0.09 | - | -^a^ | - | - |
| Bi | ^115^In | y=0.0663X + 0.2392 | QL-2000 | 0.9993 | 0.15 | - | -^a^ | - | - |
| Ca | ^45^Sc | y=0.0003X + 5.2613 | QL-2000 | 0.9994 | 820 | 51632 | 58400 ± 1900 | 88.4 | 0.34 |
| Cd | ^115^In | y=0.0045X + 0.1001 | QL-2000 | 0.9957 | 0.17 | 60.0 | 73.7 ± 2.3 | 80.9 | 0.70 |
| Co | ^89^Y | y=0.0266X + 0.0434 | QL-2000 | 0.9999 | 0.19 | 15.4 | 17.9 ± 0.68 | 86.0 | 0.43 |
| Cr | ^45^Sc | y=0.0089X + 0.2691 | QL-2000 | 0.9996 | 6.8 | 237 | 402 ± 13 | 60.6 | 4.7 |
| Cs | ^115^In | y=0.060X + 0.7257 | QL-2000 | 0.9975 | 0.62 | 2.8 | 3.4 ± 0.2 | 83.4 | 0.28 |
| Cu | ^89^Y | y=0.0143X + 0.0646 | QL-2000 | 0.9997 | 9.6 | 589 | 610 ± 70 | 96.5 | 0.13 |
| Fe | ^45^Sc | y=0.0118X + 10.074 | QL-2000 | 0.9995 | 209 | 34290 | 39200 ± 2100 | 87.1 | 0.44 |
| K | ^45^Sc | y= 0.0034X - 0.0312 | QL-2000 | 0.9999 | 96.0 | 7884 | 10560 ± 490 | 75.0 | 1.2 |
| Li | ^45^Sc | y= 0.0037X + 0.03893 | QL-2000 | 1.0000 | 5.9 | - | -^a^ | - | - |
| Mg | ^45^Sc | y=0.0021X + 0.0854 | QL-2000 | 0.9997 | 203 | 6483 | 8130 ± 20 | 79.7 | 1.0 |
| Mn | ^45^Sc | y=0.017 X+ 0.2661 | QL-2000 | 0.9997 | 3.0 | 646 | 790 ± 44 | 81.6 | 0.27 |
| Mo | ^103^Rh | y=0.0097X - 0.064 | QL-2000 | 0.9994 | 17.2 | - | -^a^ | - | - |
| Na | ^45^Sc | y= 0.0037X + 0.0664 | QL-2000 | 0.9999 | 547 | 3303 | 4240 ± 60 | 78.5 | 0.97 |
| Ni | ^72^Ge | y=1.1571X- 3.6309 | QL-2000 | 0.9998 | 1.5 | 79.3 | 81.1 ± 6.8 | 97.8 | 7.9 |
| P | ^45^Sc | y=4x10^-7^X + 4x10^-6^ | QL-2000 | 0.9997 | 76.2 | - | -^a^ | - | - |
| Pb | ^115^In | y=0.0488X + 0.1791 | QL-2000 | 0.9996 | 2.9 | 6373 | 6550 ± 330 | 96.6 | 0.68 |
| Sb | ^115^In | y=0.0167X - 0.0018 | QL-2000 | 0.9999 | 0.51 | 36.0 | 45.4 ± 1.4 | 78.5 | 0.94 |
| Se | ^72^Ge | y=0.0054X + 0.0675 | QL-2000 | 0.9981 | 3.7 | 27.7 | 28.4 ± 1.1 | 90.7 | 6.7 |
| Sn | ^115^In | y= 0.0124X + 0.0897 | QL-2000 | 0.9995 | 3.1 | - | -^a^ | - | - |
| Sr | ^89^Y | y=0.0546X + 0.0183 | QL-2000 | 1.0000 | 2.1 | 190 | 215 ± 17 | 88.3 | 0.24 |
| Tl | ^115^In | y=0.0477X + 0.1878 | QL-2000 | 0.9991 | 0.05 | - | -^a^ | - | - |
| V | ^45^Sc | y= 0.0354X - 0.0159 | QL-2000 | 0.9998 | 0.27 | 104 | 127 ± 11 | 81.1 | 0.37 |
| Zn | ^89^Y | y= 0.0032X + 0.6846 | QL-2000 | 0.9999 | 20.8 | 3947 | 4800 ± 270 | 82.2 | 0.76 |
| ^a^ Not certified | | | | | | | | | |

**STable 3.** Mean, standard deviation (SD), minimum (Min) and maximum (Max) values of PM_10_ mass and elements concentrations (µg m^‑3^), and equivalent black carbon (eBC) and total organic carbon concentrations (TOC) (ng m^-3^) found in studied samples (N=104), together with non-quantitated ratios (expressed as percentage).

|  | **Mean** | **SD** | **Min** | **Max** | **Non-quantitated**  **(%)** |
| --- | --- | --- | --- | --- | --- |
| PM_10_ mass | 41.6 | 16.0 | 16.5 | 88.7 | 0% |
| Al | 9706 | 18752 | <930 | 52922 | 4% |
| As | 2.0 | 1.2 | 0.24 | 5.3 | 0% |
| Ba | 95.1 | 155 | <7.3 | 606 | 8% |
| Be | 0.20 | 0.40 | <0.09 | 1.4 | 85% |
| Bi | 1.9 | 2.2 | <0.15 | 7.6 | 14% |
| Ca | 11357 | 20738 | <820 | 84893 | 17% |
| Cd | 0.80 | 0.51 | <0.17 | 2.0 | 6% |
| Co | 1.0 | 0.72 | <0.19 | 2.8 | 5% |
| Cr | 21.4 | 25.3 | <6.8 | 94.0 | 17% |
| Cs | 0.40 | 0.42 | <0.62 | 1.5 | 88% |
| Cu | 48.2 | 38.7 | <9.6 | 183 | 4% |
| Fe | 851 | 495 | 226 | 2834 | 0% |
| K | 1039 | 2391 | <96.0 | 12357 | 38% |
| Li | 3.5 | 4.3 | <5.9 | 17.3 | 85% |
| Mg | 7443 | 14145 | <203 | 46032 | 17% |
| Mn | 19.8 | 13.0 | <3.0 | 65.5 | 1% |
| Mo | 15.3 | 14.4 | <17.2 | 40.5 | 43% |
| Na | 7494 | 5577 | <547 | 14759 | 14% |
| Ni | 2.3 | 2.0 | <1.5 | 10.1 | 1% |
| P | 366 | 394 | <76.2 | 1552 | 5% |
| Pb | 19.5 | 14.9 | 3.7 | 68.1 | 0% |
| Sb | 5.4 | 3.4 | 0.71 | 19.3 | 0% |
| Se | 2.7 | 1.5 | <3.7 | 6.1 | 12% |
| Sn | 12.6 | 9.7 | <3.1 | 35.5 | 6% |
| Sr | 27.5 | 53.6 | <2.1 | 143 | 8% |
| Tl | 0.20 | 0.22 | <0.05 | 0.68 | 5% |
| V | 3.0 | 1.8 | 0.36 | 7.7 | 0% |
| Zn | 170 | 152 | <20.8 | 805 | 4% |
| C | 10462 | 4020 | 3280 | 16420 | 0% |
| H | 1688 | 2991 | 100 | 14730 | 0% |
| N | 770 | 333 | 270 | 1450 | 0% |
| S | 950 | 872 | <40 | 3670 | 1% |
| eBC | 3901 | 1318 | 1177 | 5447 | 0% |
| TOC | 11014 | 3925 | 3220 | 16020 | 0% |

**STable 4.** Mean, standard deviation (SD), minimum (Min) and maximum (Max) values of individual PM_10_-bound PAHs and PAHs summation (ΣPAHs) concentrations (ng m^-3^) found in samples (N=104).

| **Compound** | **Mean** | **SD** | **Min** | **Max** | **Ring number** | **Not quantified sample**  **(%)** |
| --- | --- | --- | --- | --- | --- | --- |
| NAP | 0.21 | 0.28 | <0.075 | 2.0 | 2 | 2 |
| Me-NAP | 0.39 | 1.0 | <0.039 | 7.5 | 2 | 1 |
| ACE | 0.032 | 0.043 | <0.003 | 0.34 | 3 | 8 |
| ACY | 0.018 | 0.041 | <0.005 | 0.30 | 3 | 22 |
| FLU | 0.055 | 0.091 | <0.003 | 0.69 | 3 | 1 |
| Me-FLU | 0.083 | 0.19 | <0.021 | 1.2 | 3 | 48 |
| PHE | 0.17 | 0.10 | 0.019 | 0.61 | 3 | 0 |
| ANT | 0.054 | 0.055 | <0.015 | 0.38 | 3 | 2 |
| Me-ANT | 0.018 | 0.021 | <0.004 | 0.13 | 3 | 3 |
| FLT | 0.13 | 0.083 | 0.019 | 0.41 | 4 | 0 |
| PYR | 0.18 | 0.12 | 0.027 | 0.59 | 4 | 0 |
| RET | 0.014 | 0.053 | <0.002 | 0.29 | 3 | 90 |
| BaA | 0.070 | 0.085 | <0.001 | 0.37 | 4 | 3 |
| TPY | 0.089 | 0.063 | <0.001 | 0.33 | 4 | 2 |
| CHR | 0.18 | 0.12 | 0.021 | 0.58 | 4 | 0 |
| BbjF | 0.74 | 0.50 | 0.069 | 2.7 | 5 | 0 |
| BkF | 0.19 | 0.15 | 0.016 | 0.85 | 5 | 0 |
| BeP | 0.15 | 0.13 | 0.011 | 0.76 | 5 | 0 |
| BaP | 0.28 | 0.17 | 0.040 | 0.95 | 5 | 0 |
| DahA | 0.048 | 0.11 | <0.002 | 0.52 | 5 | 61 |
| IcdP | 0.52 | 0.32 | <0.010 | 1.6 | 6 | 7 |
| BghiP | 0.83 | 0.36 | 0.093 | 1.6 | 6 | 0 |
| ΣPAHs | 3.9 | 1.9 | 0.79 | 10.8 |  |  |

**STable 5.** Benzo(a)pyrene-equivalent toxic factors (TEF) considered.

| **Compound** | **TEF** | **Reference** |
| --- | --- | --- |
| NAP | 0.001 | (Samburova et al., 2017) |
| Me-NAP | 0.001 | (Samburova et al., 2017) |
| ACY | 0.001 | (Samburova et al., 2017) |
| ACE | 0.001 | (Samburova et al., 2017) |
| FLU | 0.001 | (Samburova et al., 2017) |
| PHE | 0.001 | (Samburova et al., 2017) |
| ANT | 0.01 | (Samburova et al., 2017) |
| FLT | 0.08 | (MDH, 2014) |
| RET | 0.001 | (Samburova et al., 2017) |
| PYR | 0.001 | (Samburova et al., 2017) |
| BaA | 0.2 | (MDH, 2014) |
| CHR | 0.1 | (MDH, 2014) |
| BbF | 0.1 | (Samburova et al., 2017) |
| BeP | 0.002 | (Callén et al., 2011) |
| BaP | 1.0 | (MDH, 2014) |
| DahA | 10.0 | (MDH, 2014) |
| IcdP | 0.07 | (MDH, 2014) |
| BghiP | 0.009 | (MDH, 2014) |
| DalP | 30.0 | (MDH, 2014) |

**STable 6.** Classification of chemical variables (PMF5 model).

| **Species** | **Category** | **S/N** | **Min** | **25th** | **Median** | **75th** | **Max** | **% Modeled Samples** | **% Raw Samples** |
| --- | --- | --- | --- | --- | --- | --- | --- | --- | --- |
| PM_10_ mass | Strong | 9.0 | 16.5 | 28.0 | 35.4 | 48.1 | 88.7 | 96.3 | 100 |
| Al | Strong | 5.0 | 548 | 2026 | 2337 | 3373 | 63061 | 96.3 | 100 |
| As | Strong | 6.9 | 0.38 | 0.70 | 1.1 | 2.3 | 4.1 | 96.3 | 100 |
| Ba | Weak | 4.5 | 2.1 | 23.0 | 33.3 | 51.1 | 578 | 96.3 | 100 |
| Be | Bad | 2.5 | 0.07 | 0.07 | 0.07 | 0.07 | 1.2 | 0 | 100 |
| Bi | Strong | 6.4 | 0.01 | 0.54 | 1.2 | 1.9 | 6.1 | 96.3 | 100 |
| Ca | Strong | 6.0 | 0.0 | 309 | 559 | 6447 | 50444 | 96.3 | 100 |
| Cd | Weak | 4.5 | 0.02 | 0.25 | 0.36 | 0.82 | 2.0 | 96.3 | 100 |
| Co | Strong | 5.8 | 0.07 | 0.32 | 0.76 | 1.5 | 2.4 | 96.3 | 100 |
| Cr | Strong | 5.0 | 0.0 | 3.0 | 10.9 | 29.8 | 115 | 96.3 | 100 |
| Cs | Bad | 0.8 | 0.0 | 0.0 | 0.31 | 0.31 | 1.4 | 0 | 100 |
| Cu | Weak | 4.4 | 5.8 | 16.8 | 26.6 | 39.5 | 88.4 | 96.3 | 100 |
| Fe | Strong | 7.2 | 256 | 492 | 774 | 1093 | 1851 | 96.3 | 100 |
| K | Weak | 4.8 | 0.03 | 0.25 | 822 | 1020 | 1445 | 96.3 | 100 |
| Li | Bad | 1.4 | 3.4 | 3.5 | 3.6 | 3.6 | 13.6 | 0 | 100 |
| Mg | Strong | 6.3 | 34.9 | 127 | 2929 | 3188 | 40588 | 96.3 | 100 |
| Mn | Strong | 5.1 | 4.3 | 9.0 | 15.5 | 25.4 | 44.4 | 96.3 | 100 |
| Mo | Weak | 3.2 | 0.04 | 0.04 | 10.3 | 32.8 | 44.6 | 96.3 | 100 |
| Na | Strong | 6.5 | 322 | 335 | 10844 | 12206 | 14759 | 96.3 | 100 |
| Ni | Weak | 4.4 | 0.65 | 0.90 | 0.92 | 2.4 | 8.2 | 96.3 | 100 |
| P | Strong | 6.1 | 5.3 | 98.3 | 275 | 539 | 1.865 | 96.3 | 100 |
| Pb | Strong | 8.0 | 6.8 | 8.9 | 17.0 | 30.8 | 51.3 | 96.3 | 100 |
| Sb | Strong | 8.1 | 0.71 | 2.2 | 4.3 | 7.0 | 19.3 | 96.3 | 100 |
| Se | Bad | 1.2 | 0.52 | 2.0 | 2.2 | 2.3 | 5.7 | 0 | 100 |
| Sn | Weak | 4.7 | 0.34 | 3.1 | 6.0 | 13.5 | 43.3 | 96.3 | 100 |
| Sr | Weak | 4.4 | 1.1 | 3.9 | 6.6 | 9.3 | 171 | 96.3 | 100 |
| Tl | Bad | 2.9 | 0.0 | 0.03 | 0.06 | 0.12 | 0.33 | 0 | 100 |
| V | Strong | 8.5 | 0.44 | 1.4 | 1.9 | 3.6 | 6.8 | 96.3 | 100 |
| Zn | Strong | 5.4 | 7.2 | 37.4 | 61.1 | 143 | 364 | 96.3 | 100 |
| C | Strong | 9.0 | 4081 | 7155 | 9198 | 13585 | 19876 | 96.3 | 100 |
| H | Strong | 8.9 | 497 | 896 | 1147 | 1648 | 14415 | 96.3 | 96.3 |
| N | Strong | 8.6 | 374 | 473 | 575 | 1006 | 1341 | 96.3 | 100 |
| S | Strong | 6.7 | 50.6 | 191 | 647 | 1486 | 3808 | 96.3 | 100 |
| eBC | Strong | 9.0 | 1488 | 2717 | 3792 | 5011 | 6643 | 96.3 | 100 |
| TOC | Strong | 9.0 | 4185 | 7352 | 9904 | 13974 | 19110 | 96.3 | 100 |

**STable 7.** Mean, standard deviation (SD), minimum (Min), maximum (Max) values and range of Isotopic Variation of δ^13^C (‰) obtained for PM_10_ samples (N=104).

| **Site** | **n** | **Mean** | **SD** | **Min** | **Max** | **Range** |
| --- | --- | --- | --- | --- | --- | --- |
| MED-1 | 27 | -25.6 | 0.5 | -27.0 | -25.0 | 2.0 |
| MED-2 | 25 | -25.8 | 0.2 | -26.2 | -25.1 | 1.1 |
| ITA-1 | 27 | -25.7 | 0.4 | -26.9 | -25.0 | 1.9 |
| ITA-2 | 25 | -25.6 | 0.3 | -26.3 | -25.1 | 1.2 |
| Total | 104 | -25.7 | 0.4 | -27.0 | -25.0 | 2.0 |

**STable 8.** Spatial variation of lifetime cancer risks (LCRs) associated to metal(oid)s exposure.

| **Site** | **n** | **Mean** | **SD** | **RSD%** | **Min** | **Max** |
| --- | --- | --- | --- | --- | --- | --- |
| MED-1 | 27 | 2.8 x 10^-6^ | 1.8 x 10^-6^ | 65.7 | 5.5 x 10^-6^ | 7.1 x 10^-6^ |
| MED-2 | 25 | 3.4 x 10^-6^ | 2.2 x 10^-6^ | 64.2 | 4.4 x 10^-6^ | 9.4 x 10^-6^ |
| ITA-1 | 27 | 4.2 x 10^-6^ | 2.1 x 10^-6^ | 48.5 | 1.7 x 10^-6^ | 8.0 x 10^-6^ |
| ITA-2 | 25 | 3.2 x 10^-6^ | 1.8 x 10^-6^ | 55.1 | 4.6 x 10^-6^ | 7.1 x 10^-6^ |
| Total | 104 | 3.4 x 10^-6^ | 2.0 x 10^-6^ | 58.9 | 4.4 x 10^-6^ | 8.9 x 10^-6^ |

**SFigure 1.** Mean temporal variations of PM_10_ and PM_2.5_ mass concentrations (µg m^-3^) in Aburrá valley during 2017. Air quality Network Early warning system in the Aburrá Valley (SIATA). Source: <https://siata.gov.co/siata_nuevo/>

**SFigure 2.** Variation of precipitation (P1, mm) in Aburrá valley during 2017. Air quality Network Early warning system in the Aburrá Valley (SIATA). Source: <https://siata.gov.co/siata_nuevo/>


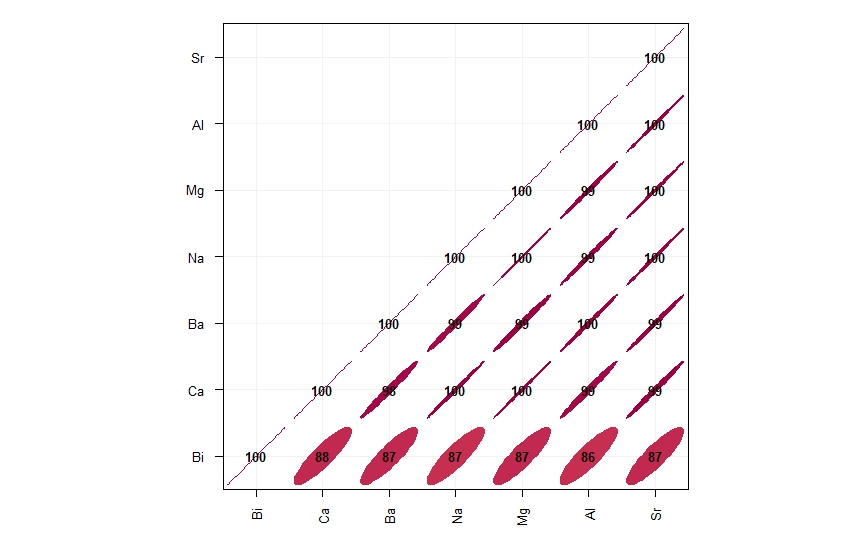


**SFigure 3.** Correlation matrix (Spearman) for elements associated to crustal origin at MED-1 site.

**SFigure 4.** Q/Qexp variation by factors at sampling sites.


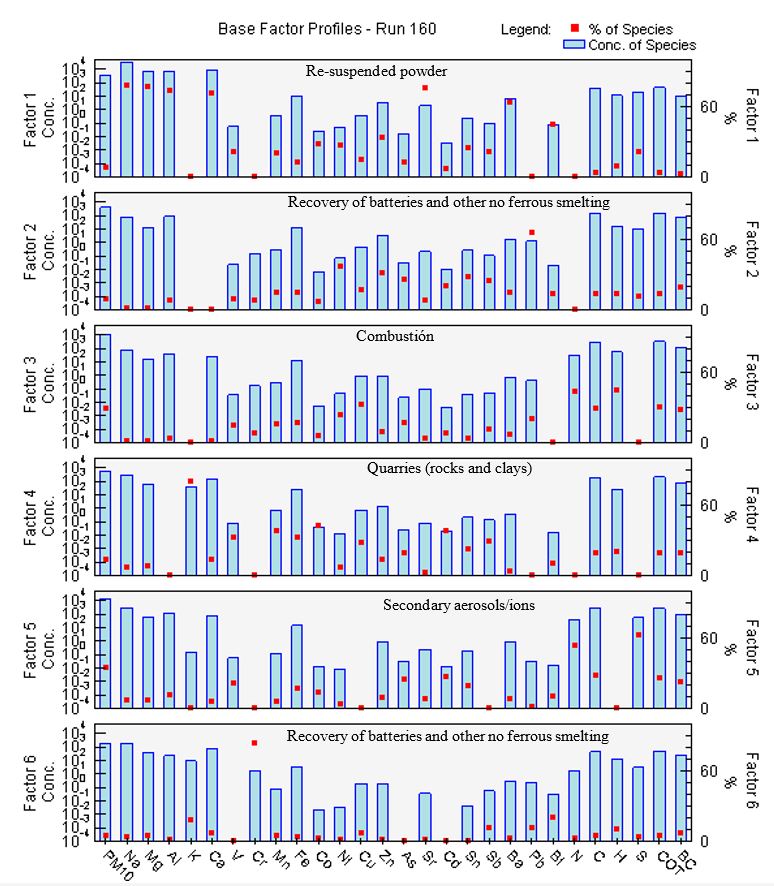


**SFigure 5-A.** Source profiles for MED-1 site by using EPA PMF 5.0 software.


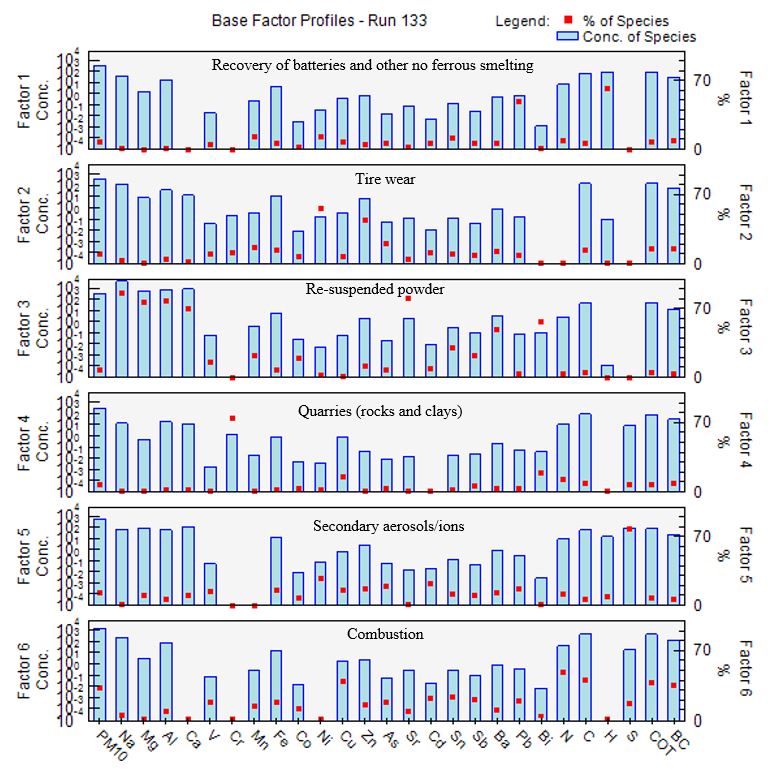


**SFigure 5-B.** Source profiles for ITA-1 site by using EPA PMF 5.0 software.


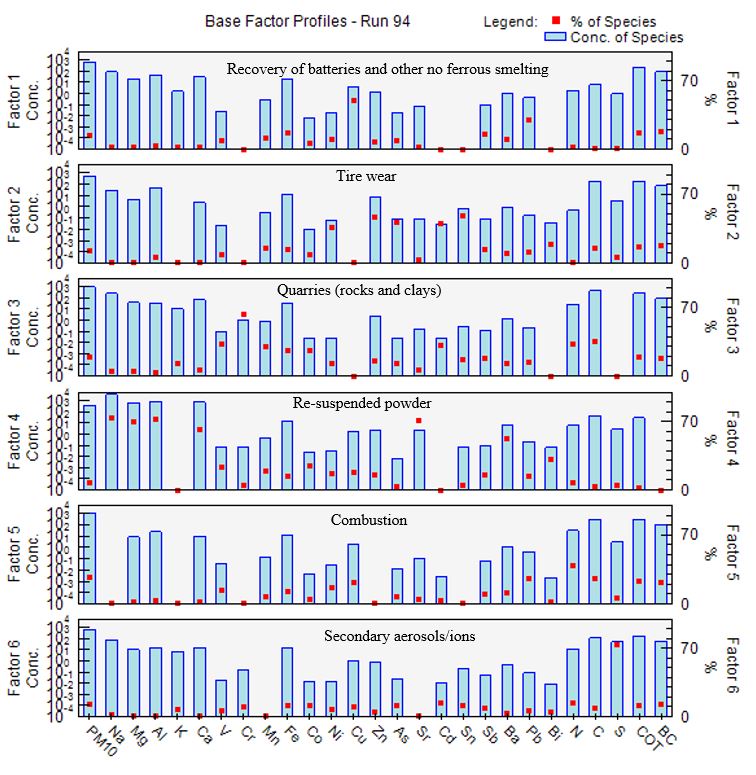


**SFigure 5-C.** Source profiles for MED-2 site by using EPA PMF 5.0 software.


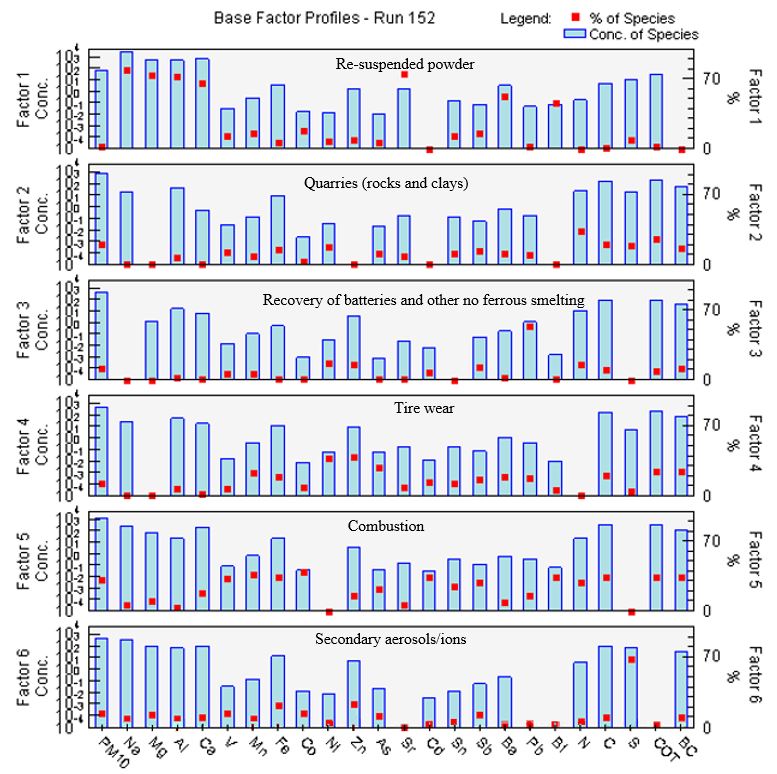


**SFigure 5-D.** Source profiles for ITA-2 site by using EPA PMF 5.0 software.

**SFigure 6.** PM_2.5_/PM_10_ mass concentrations relationship (µg m^-3^) for MED-1 site (March 2017- October 2017). Information source: [www.siata.gov.co](http://www.siata.gov.co)

**SFigure 7.** Contribution of PAHs sorted by condensed aromatic ring-numbers for each sampling site.

**SFigure 8.** Variation of Lifetime Cancer Risks (LCRs) associated to PM_10_-bound metal(oid)s exposure: spatial (A) and temporal (B) variations.

**SFigure 9.** Temporal variation of wind speed (m s^-1^) at sampling sites during 2017. Data from meteorological station UNAL Source: www.siata. gov.co

**SFigure 10.** Temporal variation of temperature (T, °C) at sampling sites during 2017. Data from meteorological station UNAL Source: www.siata. gov.co

**References**

Callén MS, De La Cruz MT, López JM, Mastral AM (2011) PAH in airborne particulate matter.: Carcinogenic character of PM_10_ samples and assessment of the energy generation impact. Fuel Process Technol 92:176–182. https://doi.org/10.1016/j.fuproc.2010.05.019

MDH (2014) Guidance for Evaluating the Cancer Potency of Polycyclic Aromatic Hydrocarbon (PAH) Mixtures in Environmental Samples. https://www.health.state.mn.us/communities/environment/risk/docs/guidance/pahguidance.pdf (accessed 09/03/2022)

Mu Y, Qin X, Liu J, Yin Z (2015) Methods Development for the Optical Determination of the Black Carbon Content of Loess Samples. Am J Anal Chem 6:585–603. https://doi.org/10.4236/ajac.2015.67057

Murray JR, Penning TM (2018) Carcinogenic polycyclic aromatic hydrocarbons. Comprehensive Toxicol 7:87–153. https://doi.org/10.1016/B978-0-12-801238-3.95691-5

Palma A, Di Capozzi F, Agrelli D, Amal C, Giordano S, Spagnuolo V, Adamo P (2018) Geochemistry and carbon isotopic ratio for assessment of PM_10_ composition, source and seasonal trends in urban environment. Environ Pollut 239:590–598. https://doi.org/10.1016/j.envpol.2018.04.064

Samburova V, Zielinska B, Khlystov A (2017) Do 16 Polycyclic Aromatic HydrocarbonsRepresent PAH Air Toxicity? Toxics 5:17. https://doi.org/10.3390/toxics5030017

USEPA (2009) Risk Assessment Guidance for Superfund Volume I: Human Health Evaluation Manual (Part F, Supplemental Guidance for Inhalation Risk Assessment). https://www.epa.gov/risk/risk-assessment-guidance-superfund-rags-part-f (accessed 3.9.22)
